# Photon Atomic Parameters of Nonessential Amino Acids for Radiotherapy and Diagnostics

**DOI:** 10.1155/2014/434519

**Published:** 2014-12-07

**Authors:** Ertuğrul O. Bursalıoğlu, Orhan İçelli, Begüm Balkan, H. Birtan Kavanoz, Mustafa Okutan

**Affiliations:** Department of Physics, Faculty of Arts and Sciences, Yıldız Technical University, Davutpaşa, 34210 İstanbul, Turkey

## Abstract

The total mass attenuation coefficients (*μ*_*t*_) (cm^2^/g) and atomic, molecular, and electronic effective cross sections have been calculated for nonessential amino acids that contain H, C, N, and O such as tyrosine, aspartate, glutamine, alanine, asparagine, aspartic acid, cysteine, and glycine in the wide energy region 0.015–15 MeV. The variations with energy of total mass attenuation coefficients and atomic, molecular, and electronic cross sections are shown for all photon interactions.

## 1. Introduction

The total mass attenuation coefficients and atomic, molecular, and electronic effective cross sections are basic quantities required in determining the penetration of X-ray and gamma photons in matter [1]. The knowledge of mass attenuation coefficients of X-rays and gamma photons in biological and other important materials is of significant interest for industrial, biological, agricultural, and medical applications [2].

Reliable data on the transmission and absorption of X-rays and gamma rays in biological, shielding, and dosimetric materials are needed in medical physics and radiation biology as well as in many other fields of medicine, biological studies, and industry. Since amino acids are the building blocks of proteins, which are essential to all living matter, data on the total attenuation cross sections of amino acids are quite useful.

Being the most abundant macromolecules that exist in living cells; amino acids constitute the largest living matter in all types of cells. The human body consists of 20 different amino acids. Out of the 20 amino acids, humans can produce 11, which are called essential amino acids. Nonessential amino acids (alanine, arginine, aspartic acid, cysteine, glutamic acid, glutamine, glycine, proline, serine, tyrosine, and asparagine) are those which can be produced from other amino acids and substances in the metabolism. The metabolism can shift into producing the amino acids that it requires for synthesizing proteins essential to our survival. Various diseases in human organism are related to amino acids. Phenylketonuria (PKU), caused by a deficiency of phenylalanine hydroxylase, is the most common clinically encountered inborn error of amino acid metabolism. Biochemically, it is characterized by accumulation of phenylalanine (and a deficiency of tyrosine). Albinism refers to a group of conditions in which a defect in tyrosine metabolism results in a deficiency in the production of melanin. These defects result in the partial or full absence of pigment from the skin, hair, and eyes. Albinism appears in different forms, and it may be inherited by one of several modes: autosomal recessive (primary mode), autosomal dominant, or X-linked. Homocystinurias are a group of disorders involving defects in the metabolism of homocysteine. The diseases are inherited as autosomal recessive illnesses, characterized by high plasma and urinary levels of homocysteine and methionine and low levels of cysteine. Alkaptonuria is a rare metabolic condition involving a deficiency in homogentisic acid oxidase, resulting in the accumulation of homogentisic acid. In addition, this reaction occurs in the degradative pathway of tyrosine [3].

The frequent and vital applications of radiation and its sources in medical and biological field requires detailed knowledge of photon mass attenuation coefficients and atomic, molecular, and electronic effective cross sections of amino acids. Investigation of radiation effects on biologically important molecules has the potential to offer insights into applications in the field of medical physics and radiation biology. For instance, electron densities are different in each tissue and they interact with external parameters such as diet, medication, environment, age, hormonal status, and genetics [4]. It has been found that normal tissue in human body exhibits the lowest values of effective electron density whereas malignant tissue the highest [4, 5]. The biologically important molecules, composed mainly of H, C, N, and O elements, carry out various physiological functions inside living systems and support production and storage of energy. Yet, depending on the quantity of the radiation absorbed by the biological matter, the extent of radiation damage is observed. In biological materials (proteins, nucleic acids, cells, and multicell organisms) very low doses are sufficient to modify and inactivate the biomolecules [6]. As seen in the literature, atomic cross sections and total dissociative electron attachment cross sections have been studied for the amino acids, glycine, alanine, proline, phenylalanine, and tryptophan, at energies below the first ionization energy by Scheer et al. [7]. Also, cross sections have been measured for two-photon absorption of aromatic amino acids by Meshalkin et al. [8].

The aim of our study is to establish a correlation between nonessential amino acids and atomic, molecular, and electronic effective cross sections and the total mass attenuation coefficients. This study is concerned with establishing whether the variations among them produce a noticeable variation in the nonessential amino acid. These cross sections also find its utilization in the computation of some other useful parameters, namely, the absorbed dose and buildup factor.

## 2. Computational Procedures

*μ*_*t*_ is the total mass attenuation coefficient and it can be given as follows:

(1) $$\begin{array}{c} \mu_{t}=\frac{\mu_{\text{linear}}}{\rho }=\left(\frac{1}{\rho \cdot x}\right)\ln\left(\frac{I_{0}}{I}\right). \end{array}$$

In this equation, *I*_0_ and *I* are the initial and final intensities of the beam passing through an absorber, *x* is the thickness, and *ρ* is the density of material, respectively. For the partial interaction processes (photoelectric absorption, pair production, Compton scattering, etc.), the index *t* is replaced with another, which is followed by the total (*t*). After determining the total mass attenuation coefficients (*μ*_*t*_) in terms of any composite (*μ*_*t*_)_ comp_ theoretically and experimentally, they can be used to define the total mass attenuation coefficients of the mixture *μ*_*t*,mix_ by the following relation:

(2) $$\begin{array}{c} \mu_{t,\text{mix}}=\sum_{i}{\left(\mu_{t,\;\text{comp }}\right)}_{i}\times w_{i}. \end{array}$$

Here, (*μ*_*t*, comp _)_*i*_ and *w*_*i*_ are the total mass attenuation coefficients and fractional weight of *i*th compound in the mixture. The total molecular effective cross section of the mixture *σ*_*t*,*m*_ can be written as

(3) $$\begin{array}{c} \sigma_{t,m}=\frac{1}{N}\mu_{t,\text{mix}}\sum_{i}{\left(\sum_{j}n_{j}A_{j}\right)}_{i}. \end{array}$$

Here, *N* is Avogadro's number and *n*_*j*_ and *A*_*j*_ are the number of atoms and molar mass of the *j*th constituent element in *i*th compound, respectively. *i* is over the all kind of compounds found in the mixture. Then, the total atomic effective cross section *σ*_*t*,*a*_ can be easily calculated from *σ*_*t*,*m*_ as

(4) $$\begin{array}{c} \sigma_{t,a}=\frac{\sigma_{t,m}}{n_{\text{total}}}. \end{array}$$

Here, *n*_total_ is the total number of atoms in the mixture chemical formula. The total electronic effective cross section *σ*_*t*,*e*_ can be expressed in the form of the following formula:

(5) $$\begin{array}{c} \sigma_{t,e}=\frac{1}{N}\sum_{i}\left(\sum_{j}\frac{f_{j}A_{j}}{Z_{j}}{\left(\mu_{t}\right)}_{j}\right)w_{i}. \end{array}$$

Here *f*_*j*_ is the molar fraction, *Z*_*j*_ is the atomic number, and (*μ*_*t*_)_*j*_ is the total mass attenuation coefficient of the *j*th element in *i*th molecule, respectively. *i* is the same as in (3).

The effective atomic numbers, effective electron density, and the total mass attenuation coefficients for nonessential amino acids can be found in the detailed formulation in the study conducted by Kavanoz et al. [9].

## 3. Results and Discussion

**Figure 1:**
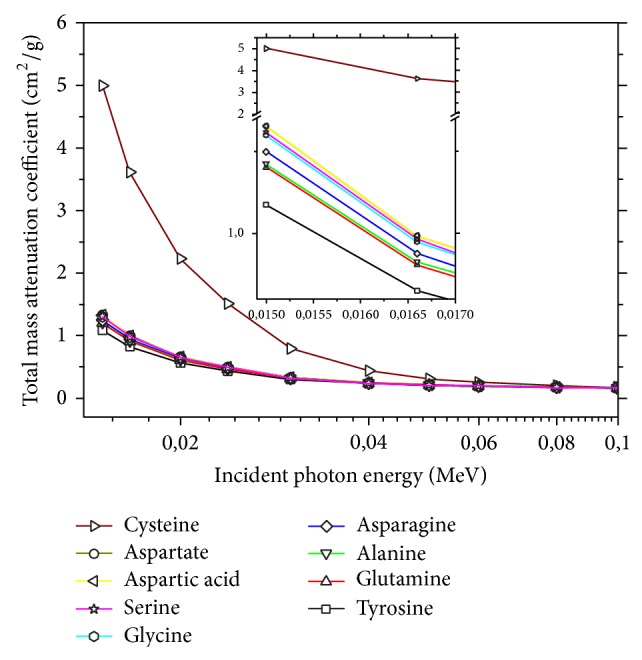
The total mass attenuation coefficients (*μ*_*t*_) of the nonessential amino acids for energy range (0.015–15 MeV).

The total mass attenuation coefficients (cm^2^/g) of gamma rays in materials and atomic, molecular, and electronic effective cross sections are of great interest for industrial, biological, agricultural, and medical studies [10]. The determined total mass attenuation coefficients of nonessential amino acids at photon energies ranging from 0.015 to 15 MeV calculated by using mixture rule from WinXCOM are shown in Figure 1 and Table 1. The total mass attenuation coefficient values of nonessential amino acids decrease with increasing photon energy. The atomic, molecular, and electronic effective cross sections are given Tables 2, 3, and 4, respectively. The atomic, molecular, and electronic effective cross sections are an important parameter in the distribution of photon flux in every object. Many researchers have studied the effective electron densities of different materials in the wide energy ranges [11–14]. Also decreasing trend with increasing photon energy is confirmed with study of Pawar and Bichile [15]. It is seen that atomic, molecular, and electronic effective cross sections of nonessential amino acids are absent in literature.

Among nonessential amino acids, tyrosine, alanine, and cysteine are known as hydrophobic. The atomic, molecular, and electronic effective cross sections decrease as the hydrophobicity increases. Cysteine is both hydrophobic and has the smallest atomic, molecular, and electronic effective cross sections. This state is confirmed by Gowda et al. [1]. Cysteine has the biggest value of atomic, molecular, and electronic effective cross sections.

Warberg first observed in 1930 that cancer cells have a fundamentally different energy metabolism from normal cells [16]. Anaerobic glycolysis causes a buildup of lactic acid to occur within the tissue. Increased tumor cell causes increasing glucose consumption, thus increase production of lactic acid (CH_3_–CH(OH)–CO(OH)). The lactic acid has a high atomic, molecular, and electronic effective cross sections compared to that in the host tissue of 8.2 × 10^23^ electrons/cm^3^ and therefore could be responsible for the increase in measured atomic, molecular, and electronic effective cross sections. There is also an increase of ketones and glutamine [17], which may also increase the overall electron density of tumor tissues. This may account for the finding that this tissue classification had a higher electron density than any other type of tissue. For nonessential amino acids with low-atomic, molecular, and electronic effective cross sections values, photons are retained; that is, they exist for a longer period of time, resulting in larger buildup value.

## 4. Conclusions

We reported new data on the total mass attenuation coefficient (*μ*_*t*_) and atomic, molecular, and electronic effective cross sections for nonessential amino acids such as tyrosine, aspartate, glutamine, alanine, asparagine, aspartic acid, cysteine, and glycine in the energy range of 0.015 to 15 MeV. In medical applications, values of the total mass attenuation coefficient and atomic, molecular, and electronic effective cross sections depend on photon energy and chemical content of amino acids in the energy regime. There is a need for more sensitive experiments to study the effect of chemical bonding on physical parameters of amino acids [18]. The novelty of the work is that the nonessential amino acids have been investigated using the total mass attenuation coefficients and the atomic, molecular, and electronic effective cross sections in the energy region 0.015–15 MeV.

The present study has been undertaken to obtain information on the total mass attenuation coefficient (*μ*_*t*_) values and atomic, molecular, and electronic effective cross sections for nonessential amino acids. It has been found that the (*μ*_*t*_) is a useful and sensitive physical quantity to determine the atomic, molecular, and electronic effective cross sections for H, C, N, and O based biological compounds.

In the interaction of photon with matter, the total mass attenuation coefficient (*μ*_*t*_) values are dependent on the physical and chemical environments of the samples. The total mass attenuation coefficient (*μ*_*t*_) values were found to decrease with increasing photon energies. Results of the study help to understand how *μ*_*t*_ values change with variation of atomic, molecular, and electronic effective cross sections values in the case of H, C, N, and O based biological compounds like nonessential amino acids.

**Figure 2:**
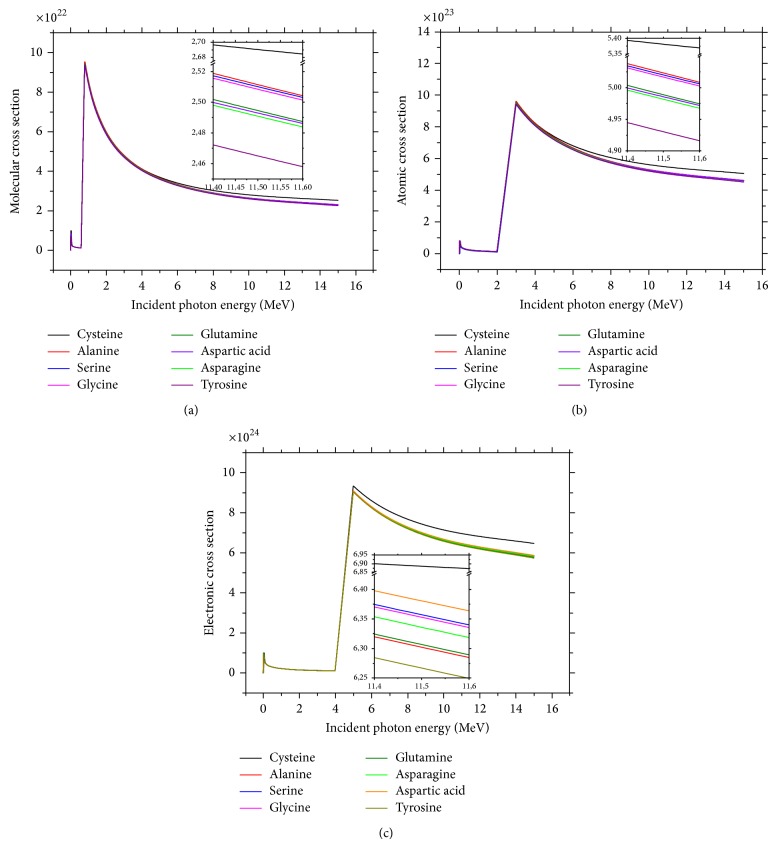
The typical plots of (*σ*_*t*,*m*_), (*σ*_*t*,*a*_), and (*σ*_*t*,*e*_) versus photon energy for nonessential amino acid: (a) *σ*_*t*,*m*_, (b) *σ*_*t*,*a*_, and (c) *σ*_*t*,*e*_.

It can be seen in Figures 2(a), 2(b), and 2(c) that atomic, molecular, and electronic effective cross sections depend on energy in 0.015 to 15 MeV energy range. There is some research which confirms this dependence [12, 13, 19, 20]. These parameters gradually decrease with increasing photon energies.

**Figure 3:**
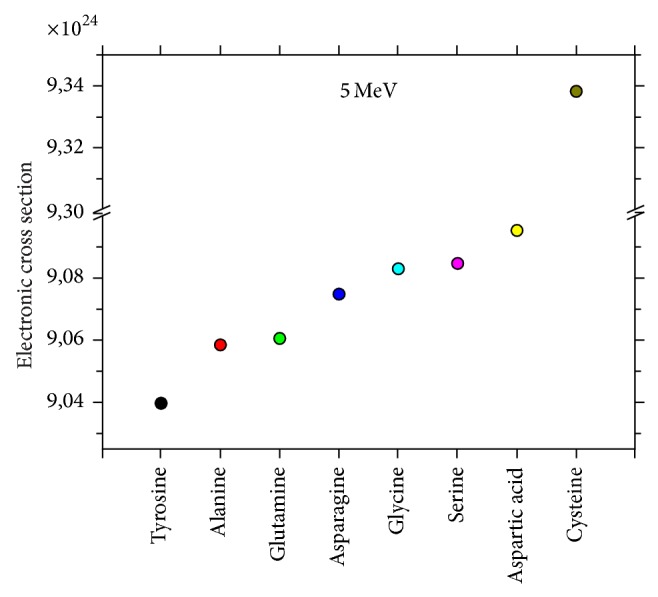
The typical plots of (*σ*_*t*,*e*_)′ versus nonessential amino acids.

As seen in Figure 3, the distributions of electronic effective cross sections were presented and correlated with nonessential amino acid types and this revealed different electronic effective cross sections values for different amino acid types. Tyrosine and cysteine have the slowest and the highest values of electronic effective cross sections, respectively.

**Figure 4:**
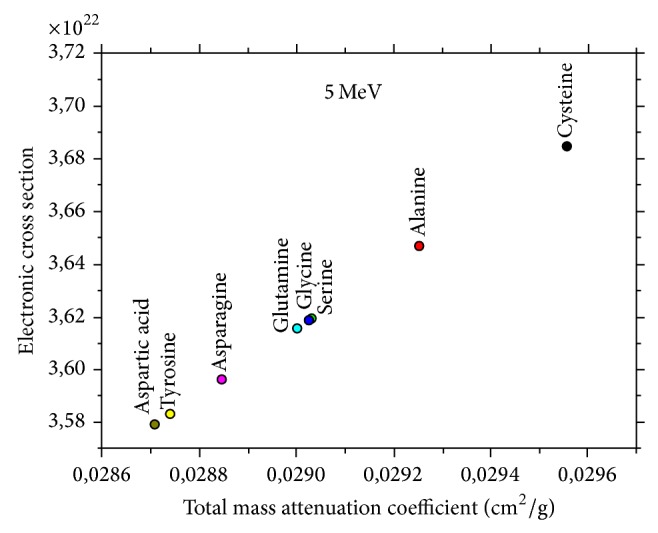
Plots of (*σ*_*t*,*e*_) as a function of (*μ*_*t*_) for nonessential amino acids.

As seen in Figure 4, linear correlation is confirmed between the total mass attenuation coefficient (*μ*_*t*_) and electronic effective cross sections. In this correlation, cysteine has more higher electronic effective cross sections than others. The photon interaction parameters would be investigated to confirm the applicability of mixture rule at different energies. It is expected that the new data on atomic, molecular, and electronic effective cross sections presented here will be useful, particularly in the energy region of interest, in view of their importance in dosimetry. Furthermore, to the best knowledge of the authors, these findings are the first of their kind at these energy levels. Because atomic, molecular, and electronic effective cross sections has the highest value among the other nonessential amino acids, cysteine shows the largest variations in the atomic, molecular, and electronic effective cross sections in the selected energy (5 MeV). Tyrosine shows the smallest variations in the atomic, molecular, and electronic effective cross sections in the selected energy (5 MeV). Therefore, atomic, molecular, and electronic effective cross sections may work as the best gamma-ray sensor from the selected nonessential amino acids [21].

**Figure 5:**
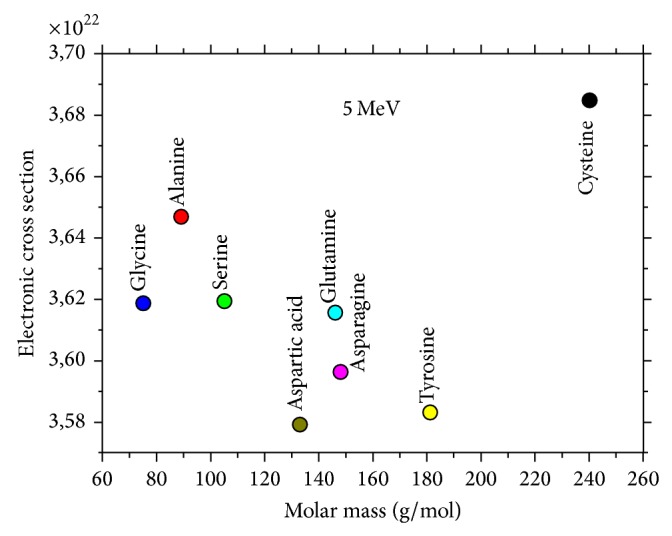
The typical plots of (*σ*_*t*,*e*_)′ versus molar mass.

As seen in Figure 5, a correlation between electronic effective cross sections and molar mass has been established. Cysteine shows the largest variations in both electronic effective cross sections and molar mass.

In view of atomic, molecular, and electronic effective cross sections it appears that atomic, molecular, and electronic effective cross sections alone are likely to be a rather poor clinical parameter for nonessential amino acids [4, 5]. However, the most important part of this study is that effective electronic cross sections ( ) may account for the finding that this amino acid classification had a higher effective electronic cross sections (*σ*_*t*,*e*_) than any other type of amino acid. A study in the future might investigate the possible interaction between atomic, molecular, and electronic effective cross sections and food consumption [22].

With this calculation study on nonessential amino acids, we hope to diagnose protein-based diseases without having to use a harmful component such as radiation. Departing from these calculations, we aim at establishing foundations related to mathematical modeling, which can be used in the diagnosis of diseases.

## Highlight

- The effective electronic cross sections and the total mass attenuation coefficients may account for nonessential amino acid classification.
- The effective electronic cross sections and the total mass attenuation coefficients may shed light on clinical parameter.
- The energy absorption in the protein may be controlled with determining parameters.

## Figures and Tables

**Table 1:** The total mass attenuation coefficients *µ*_*t*_ (cm^2^/g).

Energy (MeV)	Tyrosine	Aspartate	Glutamine	Alanine	Asparagine	Aspartic acid	Cysteine	Glycine	Serine
0.015	0.10877*E* + 01	0.13264*E* + 01	0.11995*E* + 01	0.12120*E* + 01	0.12478*E* + 01	0.13264*E* + 01	0.49934*E* + 01	0.12992*E* + 01	0.13076*E* + 01
0.020	0.56319*E* + 00	0.66003*E* + 00	0.60963*E* + 00	0.61581*E* + 00	0.62854*E* + 00	0.66003*E* + 00	0.22287*E* + 01	0.65025*E* + 00	0.65375*E* + 00
0.030	0.29844*E* + 00	0.32538*E* + 00	0.31281*E* + 00	0.31583*E* + 00	0.31730*E* + 00	0.32538*E* + 00	0.79068*E* + 00	0.32430*E* + 00	0.32530*E* + 00
0.040	0.23158*E* + 00	0.24205*E* + 00	0.23829*E* + 00	0.24055*E* + 00	0.23940*E* + 00	0.24205*E* + 00	0.43810*E* + 00	0.24292*E* + 00	0.24336*E* + 00
0.050	0.20386*E* + 00	0.20856*E* + 00	0.20792*E* + 00	0.20987*E* + 00	0.20787*E* + 00	0.20856*E* + 00	0.30915*E* + 00	0.21014*E* + 00	0.21034*E* + 00
0.060	0.18902*E* + 00	0.19143*E* + 00	0.19201*E* + 00	0.19380*E* + 00	0.19151*E* + 00	0.19143*E* + 00	0.24970*E* + 00	0.19326*E* + 00	0.19338*E* + 00
0.080	0.17214*E* + 00	0.17253*E* + 00	0.17408*E* + 00	0.17571*E* + 00	0.17321*E* + 00	0.17253*E* + 00	0.19775*E* + 00	0.17448*E* + 00	0.17453*E* + 00
0.100	0.16096*E* + 00	0.16071*E* + 00	0.16256*E* + 00	0.16408*E* + 00	0.16160*E* + 00	0.16071*E* + 00	0.17411*E* + 00	0.16268*E* + 00	0.16269*E* + 00
0.150	0.14322*E* + 00	0.14237*E* + 00	0.14429*E* + 00	0.14565*E* + 00	0.14330*E* + 00	0.14237*E* + 00	0.14707*E* + 00	0.14415*E* + 00	0.14418*E* + 00
0.200	0.13061*E* + 00	0.12984*E* + 00	0.13160*E* + 00	0.13286*E* + 00	0.13069*E* + 00	0.12984*E* + 00	0.13210*E* + 00	0.13148*E* + 00	0.13151*E* + 00
0.300	0.11336*E* + 00	0.11251*E* + 00	0.11417*E* + 00	0.11524*E* + 00	0.11335*E* + 00	0.11251*E* + 00	0.11359*E* + 00	0.11398*E* + 00	0.11398*E* + 00
0.400	0.10128*E* + 00	0.10056*E* + 00	0.10203*E* + 00	0.10299*E* + 00	0.10130*E* + 00	0.10056*E* + 00	0.10133*E* + 00	0.10188*E* + 00	0.10188*E* + 00
0.500	0.92477*E* − 01	0.91796*E* − 01	0.93150*E* − 01	0.94030*E* − 01	0.92473*E* − 01	0.91796*E* − 01	0.92381*E* − 01	0.93003*E* − 01	0.93006*E* − 01
0.600	0.85485*E* − 01	0.84857*E* − 01	0.86109*E* − 01	0.86924*E* − 01	0.85482*E* − 01	0.84857*E* − 01	0.85356*E* − 01	0.85973*E* − 01	0.85976*E* − 01
0.800	0.75122*E* − 01	0.74569*E* − 01	0.75674*E* − 01	0.76395*E* − 01	0.75121*E* − 01	0.74569*E* − 01	0.74967*E* − 01	0.75555*E* − 01	0.75558*E* − 01
1.000	0.67445*E* − 01	0.66956*E* − 01	0.67936*E* − 01	0.68577*E* − 01	0.67445*E* − 01	0.66956*E* − 01	0.67287*E* − 01	0.67832*E* − 01	0.67835*E* − 01
1.500	0.54959*E* − 01	0.54562*E* − 01	0.55364*E* − 01	0.55890*E* − 01	0.54961*E* − 01	0.54562*E* − 01	0.54834*E* − 01	0.55280*E* − 01	0.55283*E* − 01
2.000	0.47110*E* − 01	0.46802*E* − 01	0.47471*E* − 01	0.47916*E* − 01	0.47136*E* − 01	0.46802*E* − 01	0.47145*E* − 01	0.47411*E* − 01	0.47412*E* − 01
3.000	0.37777*E* − 01	0.37594*E* − 01	0.38087*E* − 01	0.38436*E* − 01	0.37838*E* − 01	0.37594*E* − 01	0.38109*E* − 01	0.38064*E* − 01	0.38065*E* − 01
4.000	0.32337*E* − 01	0.32224*E* − 01	0.32606*E* − 01	0.32897*E* − 01	0.32408*E* − 01	0.32224*E* − 01	0.32898*E* − 01	0.32603*E* − 01	0.32608*E* − 01
5.000	0.28740*E* − 01	0.28708*E* − 01	0.29001*E* − 01	0.29252*E* − 01	0.28846*E* − 01	0.28708*E* − 01	0.29556*E* − 01	0.29025*E* − 01	0.29031*E* − 01
6.000	0.26185*E* − 01	0.26203*E* − 01	0.26439*E* − 01	0.26658*E* − 01	0.26314*E* − 01	0.26203*E* − 01	0.27198*E* − 01	0.26478*E* − 01	0.26482*E* − 01
8.000	0.22817*E* − 01	0.22940*E* − 01	0.23080*E* − 01	0.23256*E* − 01	0.23003*E* − 01	0.22940*E* − 01	0.24194*E* − 01	0.23155*E* − 01	0.23156*E* − 01
10.00	0.20780*E* − 01	0.20972*E* − 01	0.21032*E* − 01	0.21183*E* − 01	0.20987*E* − 01	0.20972*E* − 01	0.22431*E* − 01	0.21132*E* − 01	0.21140*E* − 01
15.00	0.18026*E* − 01	0.18347*E* − 01	0.18291*E* − 01	0.18402*E* − 01	0.18300*E* − 01	0.18347*E* − 01	0.20201*E* − 01	0.18438*E* − 01	0.18447*E* − 01

**Table 2:** The effective atomic (σ_*t*,*a*_) cross sections for samples.

Energy (MeV)	Tyrosine	Aspartate	Glutamine	Alanine	Asparagine	Aspartic acid	Cysteine	Glycine	Serine
0.015	0.27123*E*22	0.33073*E*22	0.29910*E*22	0.30220*E*22	0.31113*E*22	0.33073*E*22	0.12451*E*21	0.32395*E*22	0.32604*E*22
0.020	0.14043*E*22	0.16458*E*22	0.15201*E*22	0.15355*E*22	0.15672*E*22	0.16458*E*22	0.55572*E*22	0.16214*E*22	0.16301*E*22
0.030	0.74415*E*23	0.81133*E*23	0.77998*E*23	0.78752*E*23	0.79118*E*23	0.81133*E*23	0.19715*E*22	0.80864*E*23	0.81112*E*23
0.040	0.57743*E*23	0.60354*E*23	0.59418*E*23	0.59980*E*23	0.59695*E*23	0.60354*E*23	0.10924*E*22	0.60572*E*23	0.60680*E*23
0.050	0.50831*E*23	0.52003*E*23	0.51845*E*23	0.52330*E*23	0.51832*E*23	0.52003*E*23	0.77086*E*23	0.52397*E*23	0.52449*E*23
0.060	0.47131*E*23	0.47733*E*23	0.47877*E*23	0.48325*E*23	0.47753*E*23	0.47733*E*23	0.62262*E*23	0.48190*E*23	0.48218*E*23
0.080	0.42923*E*23	0.43019*E*23	0.43407*E*23	0.43812*E*23	0.43190*E*23	0.43019*E*23	0.49309*E*23	0.43506*E*23	0.43519*E*23
0.100	0.40136*E*23	0.40074*E*23	0.40534*E*23	0.40912*E*23	0.40296*E*23	0.40074*E*23	0.43414*E*23	0.40563*E*23	0.40567*E*23
0.150	0.35711*E*23	0.35500*E*23	0.35979*E*23	0.36318*E*23	0.35731*E*23	0.35500*E*23	0.36671*E*23	0.35944*E*23	0.35952*E*23
0.200	0.32567*E*23	0.32376*E*23	0.32815*E*23	0.33129*E*23	0.32586*E*23	0.32376*E*23	0.32940*E*23	0.32785*E*23	0.32792*E*23
0.300	0.28267*E*23	0.28055*E*23	0.28469*E*23	0.28734*E*23	0.28263*E*23	0.28055*E*23	0.28322*E*23	0.28421*E*23	0.28421*E*23
0.400	0.25254*E*23	0.25075*E*23	0.25442*E*23	0.25681*E*23	0.25259*E*23	0.25075*E*23	0.25266*E*23	0.25404*E*23	0.25404*E*23
0.500	0.23059*E*23	0.22889*E*23	0.23227*E*23	0.23446*E*23	0.23058*E*23	0.22889*E*23	0.23035*E*23	0.23190*E*23	0.23191*E*23
0.600	0.21315*E*23	0.21159*E*23	0.21471*E*23	0.21674*E*23	0.21315*E*23	0.21159*E*23	0.21283*E*23	0.21437*E*23	0.21438*E*23
0.800	0.18731*E*23	0.18594*E*23	0.18869*E*23	0.19049*E*23	0.18731*E*23	0.18594*E*23	0.18693*E*23	0.18840*E*23	0.18840*E*23
1.000	0.16817*E*23	0.16695*E*23	0.16940*E*23	0.17100*E*23	0.16817*E*23	0.16695*E*23	0.16778*E*23	0.16914*E*23	0.16914*E*23
1.500	0.13704*E*23	0.13605*E*23	0.13805*E*23	0.13936*E*23	0.13704*E*23	0.13605*E*23	0.13673*E*23	0.13784*E*23	0.13785*E*23
2.000	0.11747*E*23	0.11670*E*23	0.11837*E*23	0.11948*E*23	0.11753*E*23	0.11670*E*23	0.11755*E*23	0.11822*E*23	0.11822*E*23
3.000	0.94197*E*24	0.93741*E*24	0.94969*E*24	0.95839*E*24	0.94349*E*24	0.93741*E*24	0.95023*E*24	0.94910*E*24	0.94914*E*24
4.000	0.80631*E*24	0.80351*E*24	0.81302*E*24	0.82027*E*24	0.80810*E*24	0.80351*E*24	0.82029*E*24	0.81296*E*24	0.81307*E*24
5.000	0.71664*E*24	0.71584*E*24	0.72314*E*24	0.72938*E*24	0.71927*E*24	0.71584*E*24	0.73696*E*24	0.72374*E*24	0.72387*E*24
6.000	0.65292*E*24	0.65336*E*24	0.65926*E*24	0.66470*E*24	0.65614*E*24	0.65336*E*24	0.67818*E*24	0.66023*E*24	0.66031*E*24
8.000	0.56893*E*24	0.57201*E*24	0.57549*E*24	0.57989*E*24	0.57358*E*24	0.57201*E*24	0.60328*E*24	0.57736*E*24	0.57740*E*24
10.00	0.51815*E*24	0.52293*E*24	0.52442*E*24	0.52819*E*24	0.52330*E*24	0.52293*E*24	0.55932*E*24	0.52692*E*24	0.52711*E*24
15.00	0.44948*E*24	0.45747*E*24	0.45608*E*24	0.45884*E*24	0.45630*E*24	0.45747*E*24	0.50370*E*24	0.45974*E*24	0.45997*E*24

**Table 3:** The effective molecular (σ_*t*,*m*_) cross sections for samples.

Energy (MeV)	Tyrosine	Aspartate	Glutamine	Alanine	Asparagine	Aspartic acid	Cysteine	Glycine	Serine
0.015	0.13561*E*21	0.16537*E*21	0.14955*E*21	0.15110*E*21	0.15557*E*21	0.16537*E*21	0.62255*E*21	0.16198*E*21	0.16302*E*21
0.020	0.70215*E*22	0.82289*E*22	0.76004*E*22	0.76776*E*22	0.78362*E*22	0.82289*E*22	0.27786*E*21	0.81069*E*22	0.81505*E*22
0.030	0.37208*E*22	0.40566*E*22	0.38999*E*22	0.39376*E*22	0.39559*E*22	0.40566*E*22	0.98576*E*22	0.40432*E*22	0.40556*E*22
0.040	0.28872*E*22	0.30177*E*22	0.29709*E*22	0.29990*E*22	0.29847*E*22	0.30177*E*22	0.54620*E*22	0.30286*E*22	0.30340*E*22
0.050	0.25415*E*22	0.26002*E*22	0.25923*E*22	0.26165*E*22	0.25916*E*22	0.26002*E*22	0.38543*E*22	0.26199*E*22	0.26224*E*22
0.060	0.23566*E*22	0.23867*E*22	0.23939*E*22	0.24162*E*22	0.23877*E*22	0.23867*E*22	0.31131*E*22	0.24095*E*22	0.24109*E*22
0.080	0.21462*E*22	0.21509*E*22	0.21703*E*22	0.21906*E*22	0.21595*E*22	0.21509*E*22	0.24654*E*22	0.21753*E*22	0.21760*E*22
0.100	0.20068*E*22	0.20037*E*22	0.20267*E*22	0.20456*E*22	0.20148*E*22	0.20037*E*22	0.21707*E*22	0.20282*E*22	0.20284*E*22
0.150	0.17856*E*22	0.17750*E*22	0.17990*E*22	0.18159*E*22	0.17865*E*22	0.17750*E*22	0.18336*E*22	0.17972*E*22	0.17976*E*22
0.200	0.16283*E*22	0.16188*E*22	0.16408*E*22	0.16564*E*22	0.16293*E*22	0.16188*E*22	0.16470*E*22	0.16392*E*22	0.16396*E*22
0.300	0.14133*E*22	0.14027*E*22	0.14234*E*22	0.14367*E*22	0.14131*E*22	0.14027*E*22	0.14161*E*22	0.14211*E*22	0.14211*E*22
0.400	0.12627*E*22	0.12538*E*22	0.12721*E*22	0.12840*E*22	0.12629*E*22	0.12538*E*22	0.12633*E*22	0.12702*E*22	0.12702*E*22
0.500	0.11529*E*22	0.11445*E*22	0.11613*E*22	0.11723*E*22	0.11529*E*22	0.11445*E*22	0.11518*E*22	0.11595*E*22	0.11595*E*22
0.600	0.10658*E*22	0.10579*E*22	0.10735*E*22	0.10837*E*22	0.10657*E*22	0.10579*E*22	0.10642*E*22	0.10719*E*22	0.10719*E*22
0.800	0.93657*E*23	0.92968*E*23	0.94346*E*23	0.95245*E*23	0.93656*E*23	0.92968*E*23	0.93464*E*23	0.94198*E*23	0.94202*E*23
1.000	0.84086*E*23	0.83476*E*23	0.84699*E*23	0.85498*E*23	0.84086*E*23	0.83476*E*23	0.83889*E*23	0.84568*E*23	0.84572*E*23
1.500	0.68520*E*23	0.68024*E*23	0.69024*E*23	0.69681*E*23	0.68522*E*23	0.68024*E*23	0.68364*E*23	0.68919*E*23	0.68923*E*23
2.000	0.58734*E*23	0.58350*E*23	0.59184*E*23	0.59739*E*23	0.58767*E*23	0.58350*E*23	0.58777*E*23	0.59109*E*23	0.59110*E*23
3.000	0.47098*E*23	0.46870*E*23	0.47484*E*23	0.47919*E*23	0.47174*E*23	0.46870*E*23	0.47512*E*23	0.47455*E*23	0.47457*E*23
4.000	0.40315*E*23	0.40175*E*23	0.40651*E*23	0.41013*E*23	0.40405*E*23	0.40175*E*23	0.41015*E*23	0.40648*E*23	0.40654*E*23
5.000	0.35832*E*23	0.35792*E*23	0.36157*E*23	0.36469*E*23	0.35963*E*23	0.35792*E*23	0.36848*E*23	0.36187*E*23	0.36194*E*23
6.000	0.32646*E*23	0.32668*E*23	0.32963*E*23	0.33235*E*23	0.32807*E*23	0.32668*E*23	0.33909*E*23	0.33012*E*23	0.33015*E*23
8.000	0.28447*E*23	0.28600*E*23	0.28774*E*23	0.28995*E*23	0.28679*E*23	0.28600*E*23	0.30164*E*23	0.28868*E*23	0.28870*E*23
10.00	0.25907*E*23	0.26147*E*23	0.26221*E*23	0.26409*E*23	0.26165*E*23	0.26147*E*23	0.27966*E*23	0.26346*E*23	0.26356*E*23
15.00	0.22474*E*23	0.22874*E*23	0.22804*E*23	0.22942*E*23	0.22815*E*23	0.22874*E*23	0.25185*E*23	0.22987*E*23	0.22999*E*23

**Table 4:** The effective electronic cross sections (σ_*t*,*e*_) for samples.

Energy (MeV)	Tyrosine	Aspartate	Glutamine	Alanine	Asparagine	Aspartic acid	Cysteine	Glycine	Serine
0.015	0.35756*E*23	0.43726*E*23	0.39418*E*23	0.39766*E*23	0.41068*E*23	0.43726*E*23	0.16574*E*22	0.42736*E*23	0.43013*E*23
0.020	0.18338*E*23	0.21600*E*23	0.19831*E*23	0.19974*E*23	0.20507*E*23	0.21600*E*23	0.73779*E*23	0.21190*E*23	0.21306*E*23
0.030	0.95551*E*24	0.10496*E*23	0.99856*E*24	0.10026*E*23	0.10181*E*23	0.10496*E*23	0.25954*E*23	0.10377*E*23	0.10410*E*23
0.040	0.73450*E*24	0.77378*E*24	0.75229*E*24	0.75393*E*24	0.76048*E*24	0.77378*E*24	0.14236*E*23	0.76865*E*24	0.77008*E*24
0.050	0.64341*E*24	0.66342*E*24	0.65255*E*24	0.65333*E*24	0.65675*E*24	0.66342*E*24	0.99559*E*24	0.66086*E*24	0.66154*E*24
0.060	0.59513*E*24	0.60742*E*24	0.60082*E*24	0.60128*E*24	0.60341*E*24	0.60742*E*24	0.79880*E*24	0.60592*E*24	0.60629*E*24
0.080	0.54078*E*24	0.54611*E*24	0.54320*E*24	0.54338*E*24	0.54433*E*24	0.54611*E*24	0.62765*E*24	0.54540*E*24	0.54558*E*24
0.100	0.50515*E*24	0.50818*E*24	0.50663*E*24	0.50670*E*24	0.50728*E*24	0.50818*E*24	0.55048*E*24	0.50786*E*24	0.50791*E*24
0.150	0.44912*E*24	0.44979*E*24	0.44924*E*24	0.44929*E*24	0.44938*E*24	0.44979*E*24	0.46336*E*24	0.44954*E*24	0.44964*E*24
0.200	0.40944*E*24	0.41008*E*24	0.40959*E*24	0.40966*E*24	0.40970*E*24	0.41008*E*24	0.41572*E*24	0.40988*E*24	0.40998*E*24
0.300	0.35537*E*24	0.35532*E*24	0.35531*E*24	0.35528*E*24	0.35532*E*24	0.35532*E*24	0.35724*E*24	0.35529*E*24	0.35528*E*24
0.400	0.31745*E*24	0.31754*E*24	0.31749*E*24	0.31748*E*24	0.31751*E*24	0.31754*E*24	0.31861*E*24	0.31752*E*24	0.31752*E*24
0.500	0.28981*E*24	0.28982*E*24	0.28979*E*24	0.28980*E*24	0.28980*E*24	0.28982*E*24	0.29042*E*24	0.28980*E*24	0.28981*E*24
0.600	0.26789*E*24	0.26790*E*24	0.26788*E*24	0.26788*E*24	0.26788*E*24	0.26790*E*24	0.26832*E*24	0.26789*E*24	0.26790*E*24
0.800	0.23537*E*24	0.23539*E*24	0.23537*E*24	0.23539*E*24	0.23537*E*24	0.23539*E*24	0.23562*E*24	0.23538*E*24	0.23539*E*24
1.000	0.21138*E*24	0.21141*E*24	0.21137*E*24	0.21137*E*24	0.21138*E*24	0.21141*E*24	0.21153*E*24	0.21139*E*24	0.21140*E*24
1.500	0.17222*E*24	0.17225*E*24	0.17222*E*24	0.17223*E*24	0.17222*E*24	0.17225*E*24	0.17235*E*24	0.17224*E*24	0.17224*E*24
2.000	0.14768*E*24	0.14781*E*24	0.14774*E*24	0.14774*E*24	0.14777*E*24	0.14781*E*24	0.14827*E*24	0.14779*E*24	0.14779*E*24
3.000	0.11854*E*24	0.11884*E*24	0.11866*E*24	0.11866*E*24	0.11874*E*24	0.11884*E*24	0.12001*E*24	0.11879*E*24	0.11879*E*24
4.000	0.10158*E*24	0.10197*E*24	0.10172*E*24	0.10170*E*24	0.10182*E*24	0.10197*E*24	0.10376*E*24	0.10188*E*24	0.10189*E*24
5.000	0.90397*E*25	0.90953*E*25	0.90606*E*25	0.90585*E*25	0.90748*E*25	0.90953*E*25	0.93382*E*25	0.90830*E*25	0.90847*E*25
6.000	0.82462*E*25	0.83110*E*25	0.82720*E*25	0.82686*E*25	0.82890*E*25	0.83110*E*25	0.86076*E*25	0.82978*E*25	0.82988*E*25
8.000	0.72027*E*25	0.72927*E*25	0.72411*E*25	0.72365*E*25	0.72645*E*25	0.72927*E*25	0.76814*E*25	0.72768*E*25	0.72773*E*25
10.00	0.65764*E*25	0.66826*E*25	0.66176*E*25	0.66129*E*25	0.66449*E*25	0.66826*E*25	0.71436*E*25	0.66602*E*25	0.66626*E*25
15.00	0.57330*E*25	0.58725*E*25	0.57878*E*25	0.57816*E*25	0.58237*E*25	0.58725*E*25	0.64706*E*25	0.58437*E*25	0.58467*E*25
